# Vigabatrin-Induced Retinal Toxicity Is Partially Mediated by Signaling in Rod and Cone Photoreceptors

**DOI:** 10.1371/journal.pone.0043889

**Published:** 2012-08-30

**Authors:** Jin Yang, Matthew C. Naumann, Yi-Ting Tsai, Joaquin Tosi, Deniz Erol, Chyuan-Sheng Lin, Richard J. Davis, Stephen H. Tsang

**Affiliations:** 1 Department of Ophthalmology, Columbia University, New York, New York, United States of America; 2 Tianjin Medical University Eye Center, Tianjin, China; 3 Herbert Irving Cancer Research Center, Columbia University, New York, New York, United States of America; National Eye Institute, United States of America

## Abstract

Vigabatrin (VGB) is a commonly prescribed antiepileptic drug designed to inhibit GABA-transaminase, effectively halting seizures. Unfortunately, VGB treatment is also associated with the highest frequencies of peripheral visual field constriction of any of the antiepileptic drugs and the mechanisms that lead to these visual field defects are uncertain. Recent studies have demonstrated light exposure exacerbates vigabatrin-induced retinal toxicity. We further assessed this relationship by examining the effects of vigabatrin treatment on the retinal structures of mice with genetically altered photoreception. In keeping with previous studies, we detected increased toxicity in mice exposed to continuous light. To study whether cone or rod photoreceptor function was involved in the pathway to toxicity, we tested mice with mutations in the cone-specific *Gnat2* or rod-specific *Pde6g* genes, and found the mutations significantly reduced VGB toxicity. Our results confirm light is a significant enhancer of vigabatrin toxicity and that a portion of this is mediated, directly or indirectly, by phototransduction signaling in rod and cone photoreceptors.

## Introduction

Vigabatrin (VGB) is an antiepileptic drug that inhibits γ–aminobutyric acid (GABA) transaminase (T), an enzyme that degrades GABA. Vigabatrin’s blockade of GABA-T activity elevates free cellular GABA activity in astrocytes [1]. As GABA is a major inhibitory neurotransmitter in the brain, VGB treatment greatly reduces seizure frequency, and many patients report being seizure-free on the medication [2]. However, a well-known side effect of this drug is retinal toxicity, which causes irreversible peripheral vision loss in 30% to 50% of adult patients [3], varying degrees of retinal atrophy, and decreased visual function in children with infantile spasms [4], [5]. As awareness of this side effect has grown, it has become common to monitor patients’ vision with visual field examinations and electroretinograms (ERG). Recently, Moskowitz et al. used electroretinography to identify worsening vision among pediatric patients taking the drug and concluded that ERG testing alone may be insufficient for this purpose [6]. In addition to the ERG, sweep visual evoked potential (VEP) testing has shown promise as a tool for monitoring pediatric patients taking vigabatrin [4], [7]. Nevertheless, the mechanism of the drug’s toxicity is still poorly understood, leading to a gap in our knowledge of how to prevent its vision loss side effect in patients.

Several studies have produced leads to a possible explanation for this phenomenon, showing a correlation between vigabatrin-induced damage and phototoxicity. Duboc et al. found that the retinal sections of rats treated with VGB at a dose of 250 mg/kg/day for 45 days began showing lesions, but only when the rats were maintained in a lighted room; their counterparts that remained in darkness showed no lesions [8]. Similarly, retinal damage was observed even with mice exposed to the normal daylight cycle [9]. Yukitoshi Izumi et al. found that the retinas of mice injected with VGB and maintained in a darkroom for 20 hours after injection remained normal, with no histologic retinal damage, which would suggest light plays the definitive role in VGB toxicity. While the significance of lesions is still unclear, maintaining animals in darkness during VGB treatment may eliminate the lesions completely from the albino mouse retina [10], [11], [12]. The results of these studies support the hypothesis that light plays a critical role in VGB retinal toxicity.

**Figure 1:**
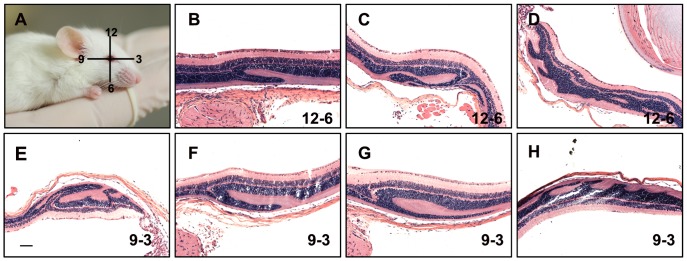
Retinal folding in two different orientations. Retinas used in this experiment are from mice exposed for 24 hours to 1500 mg/kg of VGB and kept under constant light (A). Mouse illustration shows the orientation of the section planes with respect to the position of the eye globe relative to the body. A corneal scar was introduced with a cautery pen at the 12 o’clock position. This scar, visible under a magnifying lens, can used to orient the eye globe during embedding so that the plane of section is known. (B–D) Examples of retinal folds detected along the 12–6 o’clock plane. (E–H) Retinal folds along the 9–3 o’clock plane. Scale bar: 100 µm.

Our present study attempts to confirm and extend these findings, by testing the independent role of rod and cone phototransduction in VGB retinal toxicity. We examined retinal abnormalities resulting from taking the medication in mice with mutations in *Gnat2* and *Pde6g* and showed the dependence of VGB toxicity on light. Comparisons of fold formation among the various mutant mice were used to elucidate light’s role in the mechanism of VGB retinal toxicity.

## Materials and Methods

### Mouse Lines and Husbandry

#### Ethics statement

Mice were used in accordance with the Statement for the Use of Animals issued by the Association for Research in Vision and Ophthalmology, as well as the Policy for the Use of Animals in Neuroscience Research of the Society for Neuroscience. Albino mice were used for these experiments, as they exhibit increased photoreceptor sensitivity to light compared to pigmented strains [13]. The albino MF1 strain (Harlan Laboratories) served as wild-type controls. Albino *Gnat2* mice (Jackson Laboratory) carry a mutation that extinguishes important functional domains of cone α-transducin [14], [15]. Albino W70A mice carry a null mutation in *Pde6g* and a transgene expressing a mutant PDE6g protein with a W70A substitution. They have a desensitized photoresponse, as PDE6γ is required for rod hyperpolarization [13]. The methods for creating a W70A mouse strain have been detailed elsewhere [16], [17]. Animals were housed individually and kept on a light–dark cycle (12 hour–12 hour) before the experiment. Food and water were available *ad libitum*.

**Figure 2:**
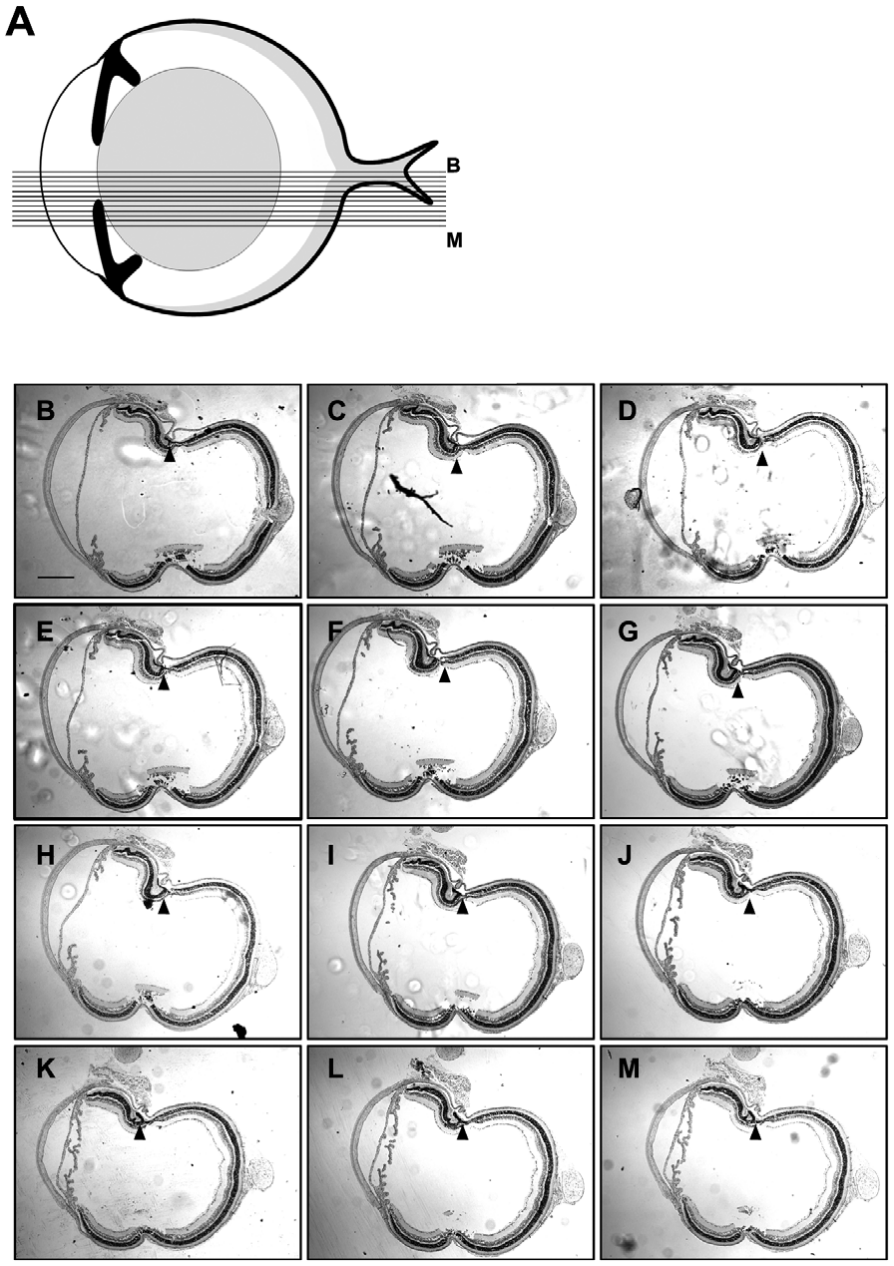
Retinal folding collected in 60 sections. Schematic of a mouse eye (A), showing the cutting pattern of the serial sections displayed from B-M. Five-micron sections were collected at regular intervals starting at the optic nerve head and continuing outwards relative the center. Approximately 60 sections were collected; every fifth section is shown here. Arrowheads indicate the position of a fold seen in all 60 sections. Retinas used in this experiment are from mice exposed for 24 hours to 1500 mg VGB/kg and exposed to a constant light source (B-M). Scale bar: 200 µm.

### Determining the Incidence of Folding in Mouse Retina

Mice were weighed to determine the volume of drug to be injected. VGB (Sabril®) was dissolved in sterile PBS and was delivered by intraperitoneal (i.p.) injection at doses ranging from 400 to 3000 mg/kg. Injections were performed when mice were between three and six weeks of age. Cages were placed in a light-proof room, maintained in a room with a 12 hour on/off light cycle, or kept under constant light exposure (5000 lux) for a predetermined period between 6–48 hours. Mice were then euthanized; eyes were enucleated and fixed in ½ Karnovsky’s fixative for 24 hours. The eyes were then embedded in paraffin and sectioned at a thickness of five microns. Approximately 10 to 12 sections were collected containing the optic nerve heads. Retinal sections were stained with hematoxylin and eosin (H&E) and were examined by light microscopy.

### Determining Folding Orientation and Size

Mice were exposed for 24 hours to VGB at 1500 mg/kg and kept under constant light (5000 lux). Before enucleation, a small scar was introduced to the cornea at the 12 o’clock position (Fig. 1A). This scar was used to orient the globe within a paraffin block with the assistance of a magnifying glass. An equal number of eyes from VGB treated mice were sectioned along the 12 to 6 o’clock and 9 to 3 o’clock axes. Retinas taken from VGB-treated mice were serial sectioned starting from the optic nerve head, proceeding at 5 micron sections for an additional 60 sections to collect ∼300 microns of retina (Fig. 2A). These sections were collected on 7.5 cm×5 cm slides.

**Figure 3:**
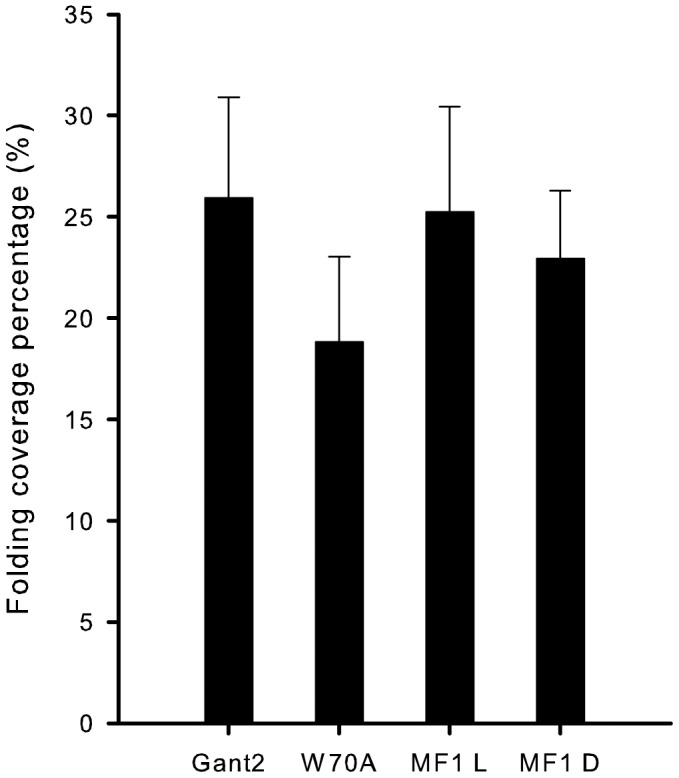
Folding coverage percentage in different mutated mice. Folding coverage percentages measured in the slides of folding-positive mice were compared between groups of mutant mice by t-test. No significant differences were found between each group. (t = 0.09, p>0.05 for *Gnat2* and MF1-light; t = 0.56, p>0.05 for W70A and MF1-light; t = 0.71, p>0.05 for *Gnat2* and W70A). (MF1L: MF1 mice kept in light; MF1D: MF1mice kept in dark).

### Statistical Analysis

A statistical analysis of the results was performed using the Statistical Package for the Social Sciences. The Chi-squared 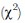 test was used to compare the incidence of the fold formation in *Gnat2* and *Pde6g*-W70A mice with MF1 mice. A p-value of p<0.05 was taken as a criterion for statistical significance.

The lengths of folds were measured and expressed as a percentage comparing fold length to the total length between the optic nerve and the ciliary body. We tested only in folding-positive mice (mice that displayed folds) (Fig. 3). The slides were serial sectioned and slides displaying the lengthiest folds were selected for measurement. Group results were expressed as the means of individual percentages ± SEM. Statistical comparisons between mutant mouse groups were performed by t-test.

**Figure 4:**
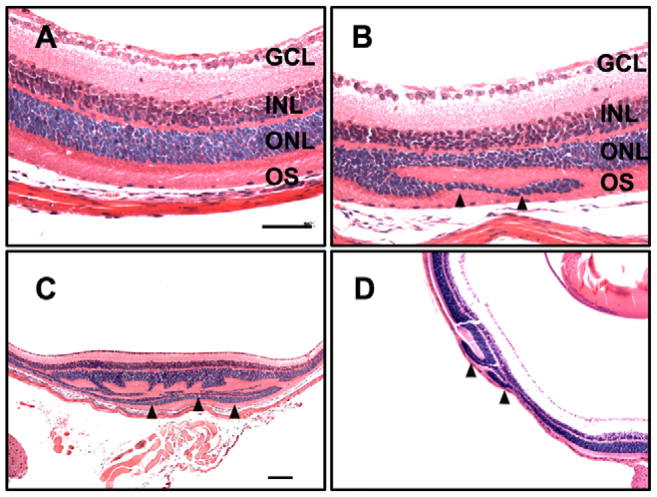
Retinal folding after VGB treatment. Retinas taken from mice injected with saline (A) or VGB at 1000 mg/kg (B-D). The ONL appears to “fold” over itself (arrowheads) and includes the OS layer. The appearance and extent of the folds can vary; compare with B, C, and D. GCL: ganglion cell layer; INL: inner nuclear layer; ONL: outer nuclear layer. Scale bars: 100 µm.

## Results

In keeping with previous studies, in which single doses of up to 1000 mg/kg have been administered to test toxicity effects in the mouse retina [9], [18], we used an expanded VGB dosage range, from 400 mg/kg to 3000 mg/kg. These higher doses, together with a light intensity of 5000 lux, were expected to result in elevated VGB levels, thus significant decreases in GABA transaminase activity and significant increases in GABA levels as soon as 2 hours after intraperitoneal injection [9], [18], [19]. Our pilot experiment using MF1 mice involved a range of VGB doses (400, 600, 800, and 1000 mg/kg). Examination of H&E stained retinas from this series demonstrated abnormal retinal structure in two of the four retinas at the 1000 mg/kg dose. This effect was restricted to the outer nuclear layer, in which a portion of the ONL folded back on itself (Fig. 4). The retinas from lower VGB doses appeared normal. Next we tested higher VGB doses ranging from 1000 to 3000 mg/kg under various light exposure durations and conditions (Table 1). Doses greater than 2000 mg/kg were found potentially lethal, but lower doses were not associated with death 24 hours after injection. Only non-lethal doses of 1500 mg/kg were used in the mice examined for retinal folding. Examination of retinal sections in pilot experiments demonstrated ONL folding in 5 of 35 cases.

**Table 1:** Pilot study under various conditions.

VGB Dose (mg/kg)	Light	Exposure (hours)	Mouse	Total eyes	Negative	Positive	Comment
1000	cycle	24	2	4	4	0	
1000	light	24	4	8	8	0	
1000	light	12	1	2	2	0	
1000	light	6	1	2	2	0	
1000	dark	12	1	2	2	0	
1000	dark	6	1	2	2	0	
1500	cycle	24	2	4	3	1	
1500	light	24	2	4	3	1	
2000	cycle	24	2	4	3	1	1 died overnight
2000	light	24	2	4	4	0	1 died overnight
2000	cycle	12	1	2	2	0	
2000	light	12	1	2	2	0	
3000	cycle	24	1	2	2	0	1 died overnight
3000	light	24	1	2	2	0	1 died overnight
3000	light	12	2	4	3	1	
3000	dark	12	1	2	1	1	

**Figure 5:**
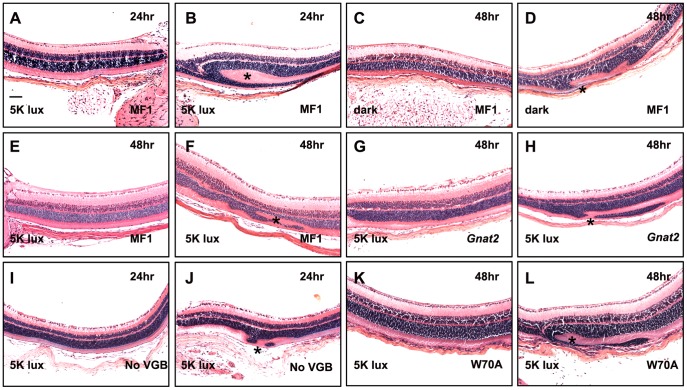
Examples of retinal sections under various conditions in different mutant mice. Panels show retinas taken from the experiments described in Tables 2 and 3. The frequencies of folding are indicated in the tables. The experimental conditions for each sample are indicated in each panel. Panels B, D, F, H, J, and L show folds (*). Panels A, C, E, G, I, and K show retinas with no detectable folds; samples are shown at the same magnification. Scale bar: 100 µm.

Since both the frequency of VGB-induced folding and the numbers of mice tested for each variable were low, we selected a single condition and increased the number of mice in an attempt to establish a meaningful folding frequency. Therefore, we treated 10 mice at a 1500 mg/kg dose, exposing these mice to constant light (5000 lux) for 24 hours (Table 2). The same number of control mice were injected with saline, exposed to constant light, and harvested at 24 hours. In this experiment, we found VGB-associated folding occurred at a rate of 35% with 7 of the 20 mouse retinas displaying folding. We observed one instance of folding in the control untreated animals, which may have been due to light exposure alone (Table 2, Fig. 5J).

**Table 2:** Incidence of VGB-associated folding at the 1500 mg/kg dose.

VGB Dose (mg/kg)	Exposure (hours)	Light (lux)	Mouse	Total eyes	Negative	Positive	Frequency
NO DRUG	24	5000	MF1	16	15	1	6%
1500	24	5000	MF1	20	13	7	35%
1500	48	5000	MF1	24	11	13	54%

To determine whether the rate of detection was influenced by folds’ small size or region-specificity, we performed orientation and serial sectioning experiments. For the orientation experiment, retinas were sectioned along either the 12 to 6 o’clock or 9 to 3 o’clock axes, with a roughly equal number of eyes with folds occurring in each group (Fig. 1). For the sectioning experiments, four retinas were sectioned completely through 300 microns, starting at the edge of the optic nerve head and continuing outwards away from the center (Fig. 2A). One of the four retinas in this test condition exhibited a fold, which was detectable in all 60 sections (Fig. 2B–M). The results suggest the orientation and the size of folds were not the reasons for low incidences of folding; the folds’ locations were independent of both the eye globe orientations and the planes of section.

Next, we tested whether extending the time of light exposure between drug injection and analysis would increase the frequency of fold detection. We injected 12 mice with 1500 mg/kg VGB and maintained them under constant light exposure (5000 lux) for 48 hours. Under these new conditions, we found that the incidence of retinal folding increased from 35% to 54% (Table 2). We next examined whether light exposure could influence vigabatrin toxicity. Mice given the standard 1500 mg/kg VGB dose but kept for 48 hours in a light-proof room showed a reduced folding incidence of 30% (dark), as compared to the control of 54% (light) (Table 3). These results suggest prolonged light exposure exacerbates VGB-induced retinal toxicity.

**Table 3:** The incidence of retinal folding in different mice.

VGB Dose (mg/kg)	Exposure (hours)	Light (lux)	Mouse	Total eyes	Negative	Positive	Frequency
1500 *	48	5000	MF1	24	11	13	54%
1500	48	Dark	MF1	23	16	7	30%
1500	48	5000	*Gnat2*	38	29	9	24%
1500	48	5000	W70A	48	38	10	21%

* Data from the 48-hour exposure under 5000 lux light experiment, shown in Table 2, is shown again here for comparison.

To extend these findings, we next tested if genetic determinants of cone or rod phototransduction play a role in VGB toxicity by analyzing VGB-treated *Gnat2* and *Pde6g*-W70A transgenic mice (Table 3, Fig. 5J, H, K, L). Similarly to the dark-exposed mice, *Gnat2* mutant mice, which completely lack α-transducin function in cone phototransduction [15], showed a reduced folding incidence (24%) compared to their counterparts the MF1 wild-type mice (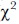
= 5.97, p<0.05). Likewise, the incidence of folding in *Pde6g*-W70A transgenic mice, which have abnormal rod phototransduction activity, was significantly reduced relative to controls (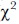
= 8.18, p<0.01). Figure 3 summarizes the folding coverage percentages among the folding-positive groups of mice. Comparisons of the four mouse groups by t-tests revealed no significant differences in folding coverage percentages among the folding-positive mice belonging to all four groups (Fig. 3) (t = 0.09, p>0.05 for *Gnat2* and MF1-light; t = 0.56, p>0.05 for W70A and MF1-light; t = 0.71, p>0.05 for *Gnat2* and W70A).

## Discussion

Vigabatrin can induce structural changes in the retina. The drug has been demonstrated to cause movement of photoreceptor nuclei toward the retinal pigment epithelium in several studies [8], [19]. These previous studies are consistent with our observation of folds in the outer nuclear layer of the retina. Although the formation of folds is closely related to vigabatrin dose [21] and duration of light exposure [9], [11], we observed that genetic mutations could also influence fold efficiency. In this study, we tested mice that carried mutations that affected rod or cone photoreceptor signaling and observed decreased folding formation.

Light has been thought to be an important factor in influencing VGB toxicity. In this study, the folding efficiency of MF1 mice kept in the dark was significantly lower than that of the light-exposed group. One mouse developed retinal folding after sustained light exposure, even without VGB injection. These results are consistent with the findings of Yukitoshi et al. [9], who reported that light is necessary for acute VGB retinotoxicity. Photoreceptor displacement was also observed in previous studies [11], [20]. Our research extended these findings: mice maintained in light conditions for 48 hours displayed increased folding incidence compared with those maintained in light conditions for 24 hours. These results suggest drug-induced retinal toxicity will occur in lighting conditions, but will be inhibited when exposure to light is limited.

VGB-elicited photoreceptor degeneration has been reported to be light-dependent by several studies [9], [11], [12]. Our results (Table 3) showed that engineered mice carrying mutations in phototransduction genes interfering with light transduction showed a decreased incidence of folding formation when given VGB. These results suggest light-induced PDE activation in rod or cone photoreceptors contributes to vigabatrin toxicity.

Considering this light dependence, we speculate rod or cone phototransduction pathway genes thus play an important role in vigabatrin toxicity. Phototransduction begins at photoreceptor outer segments [22]; activation of rhodopsin in rods and cones by light occurs, followed by stimulation of G-protein-coupled receptors that activate phosphodiesterase (PDE) [23]. Our study utilized W70A-strain and *Gnat2* mutant mice, which carry phototransduction signaling defects in their rods or cones. The W70A mice used in this study carried a point mutation in the gene encoding for the γ-subunit of rod cGMP phosphodiesterase (PDE6γ). The *Gnat2* mutant mice have extinguished important functional domains of the G-protein α-subunit [19], which has been shown to interact with all cone opsins and phosphodiesterase α-subunits [25] and to play an important role in transmitting visual signals [24]. Our findings are consistent with the interpretation that vigabatrin-induced retinal toxicity is dependent on phototransduction signaling. Interestingly, once folds had formed, their relative size and coverage did not differ among different groups of mice (Fig. 3). More investigation is needed to establish how the visual pathway is subject to interference from vigabatrin, and how these folds form in response to the toxicity.

Connecting some hypotheses which have been developed in the past about the mechanisms of VGB-induced retinal toxicity, we would consider the possibility that the drug may cause retinal toxicity through improper depolarization of the cells. In the retina, GABA is an established inhibitory neurotransmitter associated with several different types of cells [26]. Three different groups of GABA receptors are distributed in retina, termed GABA_A_, GABA_B_, and GABA_C_. GABA_A_ receptors are distributed in all neurons except for rod photoreceptors, while GABA_C_ receptors gate the Cl-channels located in cone photoreceptors and bipolar cells. GABA_C_ receptors are more sensitive to GABA than are GABA_A_ receptors. The highest concentration of these receptors is on axon terminals of rod bipolar cells [27], where they form a positive feedback loop with E_cl_, increasing the sensitivity of the mechanism that controls release of GABA and leads to cells’ depolarization. VGB elevates GABA concentration, keeping cells depolarized for longer periods of time.

In the normal phototransduction pathway, light strikes the photoreceptor, activating PDE6, diminishing the supply of cGMP, hyperpolarizing the photoreceptor cell, and causing less glutamate to be released. Then ON bipolar cells become depolarized [23]. Excessive or prolonged depolarization of these cells induces a potentially cytotoxic calcium influx [28]. The mice with mutations in the *Gnat2* or *Pde6g* genes would have reduced photoreceptor hyperpolarization, leading to reduced rates of ON bipolar cells’ depolarization. We speculate that the mutant mice are protected from retinal toxicity in this manner. The precise relationship between fold formation and cells’ depolarization is unclear but presumably involves additional mechanisms.

In summary, this study reports on VGB-elicited retinal toxicity in mice with different phototransduction mutations that have not been previously described, and gives insight into the mechanisms of light-mediated retinal toxicity. Our findings suggest that phototransduction is a significant enhancer of vigabatrin toxicity. More experiments should be done to confirm these results before they can be applied to patients who are treated with VGB.
